# Prevalence and Risk Factors Associated with Human *Taenia Solium* Infections in Mbozi District, Mbeya Region, Tanzania

**DOI:** 10.1371/journal.pntd.0002102

**Published:** 2013-03-14

**Authors:** Gloria Mwanjali, Charles Kihamia, Deodatus Vitalis Conatus Kakoko, Faustin Lekule, Helena Ngowi, Maria Vang Johansen, Stig Milan Thamsborg, Arve Lee Willingham

**Affiliations:** 1 School of Public Health and Social Sciences, Muhimbili University of Health and Allied Sciences, Dar es Salaam, Tanzania; 2 Faculty of Agriculture, Sokoine University of Agriculture, Morogoro, Tanzania; 3 Faculty of Veterinary Medicine, Sokoine University of Agriculture, Morogoro, Tanzania; 4 Section for Parasitology, Health and Development, Department of Veterinary Disease Biology, Faculty of Health and Medical Sciences, University of Copenhagen, Frederiksberg, Denmark; University of Queensland, Australia

## Abstract

**Background:**

*Taenia solium* cysticercosis/taeniosis is emerging as a serious public health and economic problem in many developing countries. This study was conducted to determine prevalence and risk factors of human *T. solium* infections in Mbeya Region, Tanzania.

**Methods and Findings:**

A cross-sectional survey was conducted in 13 villages of Mbozi district in 2009. Sera of 830 people (mean 37.9±11.3 years (SD); 43% females) were tested for circulating cysticerci antigen (Ag-ELISA) and antibody (Ab-ELISA). A subset of persons found seropositive by Ag-ELISA underwent computed tomography (CT) scan of the brain for evidence of neurocysticercosis. Stool samples from 820 of the same participants were tested for taeniosis by copro-antigens (copro-Ag-ELISA) and formol-ether concentration technique. Cases of *T. solium* taeniosis were confirmed serologically by EITB assay (rES38). A questionnaire was used for identification of risk factors. Active cysticercosis by positive Ag-ELISA was found in 139 (16.7%) persons while anti-cysticercal antibodies were detected in 376 (45.3%) persons by Ab-ELISA. Among 55 persons positive for Ag-ELISA undergoing CT scan, 30 (54.6%) were found to have structures in the brain suggestive of neurocysticercosis. Using faecal analysis, 43 (5.2%) stool samples tested positive for taeniosis by copro-Ag-ELISA while *Taenia* eggs were detected in 9 (1.1%) stool samples by routine coprology. Antibodies specifically against adult *T. solium* were detected in 34 copro-Ag-ELISA positive participants by EITB (rES38) indicating *T. solium* taeniosis prevalence of 4.1%. Increasing age and hand washing by dipping in contrast to using running water, were found associated with Ag-ELISA seropositivity by logistic regression. Gender (higher risk in females) and water source were risk factors associated with Ab-ELISA seropositivity. Reported symptoms of chronic severe headaches and history of epileptic seizures were found associated with positive Ag-ELISA (p≤0.05).

**Conclusion:**

The present study indicates *T. solium* infection in humans is highly endemic in the southern highlands of Tanzania.

## Author Summary

Cysticercosis caused by the zoonotic pork tapeworm, *Taenia solium*, is emerging as a serious public health and agricultural problem in sub-Saharan Africa. Surveys have shown cysticercosis in pigs to be highly prevalent in multiple foci in Tanzania, and a hospital-based study in the northern highlands indicated neurocysticercosis as an important cause of epileptic seizures in humans. We present here a cross-sectional community-based survey on the prevalence and risk factors of human cysticercosis and taeniosis conducted in the southern highlands – the major pig-producing area of the country. The most striking findings were that more than 15% of people surveyed were found to have active cysticercosis and nearly half of them were found to have been exposed to larval *T. solium* indicating a high level of environmental contamination with *T. solium* eggs. This was supported by finding over 4% of people having had *T. solium* tapeworms. A subset of persons found positive serologically for active cysticercosis underwent brain scanning and more than half of them were found to have neurocysticercosis. This strong evidence that *T. solium* cysticercosis/neurocysticercosis/taeniosis is highly endemic in the southern highlands of Tanzania demands urgent attention of regional and national authorities to combat the parasite.

## Introduction

Taeniosis and cysticercosis are different stages of *Taenia solium* infection involving swine as intermediate hosts and humans as definitive and/or intermediate hosts. Taeniosis is the intestinal infection with the adult cestode in humans and is caused by consumption of raw or undercooked pork containing viable cysticerci of *T. solium*. Ingestion of infective eggs passed by a person with taeniosis either by autoinfection, direct contact with another tapeworm carrier or indirectly via ingestion of contaminated food, water, or hands may also lead to cysticercosis in humans whereby larval tapeworm cysts develop in the muscles, eye and central nervous system. Pigs get cysticercosis by ingesting *T. solium* eggs primarily as a result of eating feces of a human tapeworm carrier. Human cysticercosis causes a variety of neurological symptoms, most commonly seizures due to cysts in the brain, a condition known as neurocysticercosis [1], [2].

Cysticercosis imposes substantial global burden on human beings related to epilepsy, ocular disorders, other neurological manifestations, and economic losses related to disability and lost productivity [3], [4]. A World Health Organization (WHO)-commissioned systematic review of studies reporting the frequency of neurocysticercosis (NCC) worldwide done by [5] estimated the proportion of NCC among people with epilepsy of all ages to be 29.0% (95% CI: 22.9%–35.5%), indicating epilepsy is consistently associated with NCC in over one quarter of patients residing in regions where *T. solium* infections are endemic. In sub-Saharan Africa, people with cysticercosis have been estimated to have a 3.4. to 3.8-fold increased risk for developing epilepsy [6]. These estimates confirm the importance of NCC infection in the etiology of epilepsy in developing countries and suggest that NCC may be associated with a very large burden in cysticercosis endemic areas where epilepsy is prevalent, that is, those countries where pork consumption occurs, pigs are managed under free-range conditions, and sanitation is poor or absent enabling pigs to have access to human feces [5]. Although the recognition of its status as a serious and emerging threat to public health in Africa is increasing [7] (WHO, 2010), the data on incidence in humans is very limited in most endemic areas due to a lack of adequate surveillance, monitoring and reporting systems [8].

In Tanzania *T. solium* is considered widespread in the northern, central, and southern regions based on porcine cysticercosis surveys [9], [10], [11], [12], [13]. These surveys provide initial evidence that *T. solium* infection is of national importance. Despite the high prevalences of porcine cysticercosis reported in different areas of the country, the presence of human *T. solium* infection has only been confirmed at one hospital in Mbulu district, Manyara region in the northern highlands where 13.7% of patients with epilepsy were detected to have probable/definitive NCC based on brain scans using computerised tomography and western blot to detect antibodies to larval *T. solium* [14]. However, there is still a lack of information on the *T. solium* situation (both taeniosis and cysticercosis) in the general human population [15], thus the basis for conducting the present cross-sectional survey with the aim of establishing the prevalence and identifying risk factors associated with the transmission of human *T. solium* infections in rural communities in the southern highlands of Tanzania which is the main pig raising area of the country [16].

## Methods

### Ethical statement

The study was approved (No. HD/MUH/T. 75/07) by the ethical committee at Muhimbili University of Health and Allied Sciences (MUHAS). Permission to conduct the study in the selected villages was obtained from regional, district and local authorities. Consent for selection of a participant in a sampled household was sought from the selected individual as well as the head of the household. Before interview and sample collection, each selected participant was approached individually to obtain written informed consent. For minors (<18 years) informed assent to participate in the study was obtained orally, and thereafter a written informed consent for the minor's participation was signed by a parent or guardian. Participants who were to undergo CT scanning were informed about the whole procedure and were scanned only after obtaining their written consent.

Participants with a history of epileptic seizures, testing positive by Ag-ELISA and/or detected with cysts in their brain were admitted to Vwawa District Hospital and treated according to standard of care [17]. Participants with taeniosis were treated with a single dose of Niclosamide 2 g orally. For ascariasis and hookworm infections, a single dose of Albendazole 400 mg orally was used while a single dose of Praziquantel 40 mg/kg was used only for participants with intestinal schistosomiasis who were seronegative by Ag-ELISA. The anthelmintic treatment was administered by the researcher (a nurse) under supervision of a medical doctor. No adverse effects were reported during or after anthelmintic treatment.

### Study area and population

The survey was conducted in 13 randomly selected villages in Mbozi district, Mbeya Region following a preliminary study in the district which reported a high prevalence of porcine cysticercosis (32%) based on serum Ag-ELISA (B158/B60) in 300 pigs in 150 households (Eric Komba, personal communication). The district is located in the southern highlands of Tanzania in the southwest of Mbeya region between latitudes 7° and 9° 12′ south of the equator, longitudes 32°7′30″ and 33°2′0″ east of Greenwich Meridian with altitudes between 900–2750 meters above sea level. According to the National Bureau of Statistics (http://www.nbs.go.tz), the human population of Mbozi district was 515,270 inhabitants in 2002. Inhabitants mainly engage in agriculture and keep pigs as a source of income. The total number of pigs in Mbozi district in the year 2009 was estimated to be 25,355 (District Veterinary Officer, Mbozi District - personal communication).

### Study design and sampling

A community-based descriptive cross-sectional study was conducted between April and July 2009. Sample size estimation was calculated using the formula 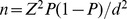 [18], in which n = required sample size, Z = Z score for a given confidence level, P = expected prevalence or proportion, and d = precision. With an estimated prevalence of 9% for taeniosis based on a Nigerian study ([19], the sample size was calculated at 385 households (one person per household). To adjust for potential non-compliance and design effect, the sample size was more than doubled such that 900 households were targeted during the survey.

A cluster-random sampling with Probability Proportion to Size (PPS) technique was used for selection of households according to [20]. In each village, the principal investigator conducted a meeting and explained the purpose of the study to the local authorities and villagers, after which households for inclusion in the survey were selected on the same day. During the following days, the research team visited the selected households and identified eligible household members. Criteria of eligibility were living in the household and being between 15–60 years old. Among all eligible household members who agreed to participate in the study only one person was selected per household by simple random sampling for questionnaire administration and collection of blood and stool samples.

After each person was interviewed, 10 ml of venous blood was drawn from cephalic or median cubital vein (median basilic vein) by medical laboratory technicians in a labelled plain blood vacutainer tube and allowed to clot at room temperature. The sera were obtained by centrifugation at 3200 rotations per min for 5 min, then aliquoted into 2 ml cryovials and stored at −20°C until tested. Similarly each participant was instructed on how to collect a stool sample and given a stool container which they returned with the sample on the same or following day. All stool samples were fixed with 10% formalin by the researcher and stored at room temperature until tested.

### Questionnaire survey

A questionnaire based on that of the Cysticercosis Working Group of Eastern and Southern Africa (www.cwgesa.dk/CWGESA/Action Plan/Research.aspx) was used to collect information on risk factors and other related information from enrolled participants. The questionnaire addressed demography, pork consumption habits, hygienic and sanitary practices, presence of subcutaneous nodules and history of neurological signs. Responses on hygienic and sanitary practices were confirmed by observation method.

The information regarding clinical signs was obtained by asking about the participants' previous histories of subcutaneous nodules, severe chronic headaches, loss of consciousness, epileptic seizures or partial seizures. According to the International League Against Epilepsy's Commission on Epidemiology and Prognosis definitions, epilepsy was defined as recurrent (two or more) epileptic seizures, unprovoked by any immediate identified cause while partial seizures were defined as presence of seizures without impairment of consciousness or awareness, *i.e.* maintained alertness and ability to interact with the environment.

### Laboratory analysis

Serological analysis was carried out using a monoclonal antibody-based ELISA detecting circulating antigens of *T. solium* cysticerci (Ag-ELISA (B158/B60) [21] and an ELISA detecting anti-cysticercal antibodies (Ab-ELISA (rT24h) [22].

Faecal studies were carried out using a copro-Ag-ELISA detecting antigens of adult *Taenia* species [23]. In addition, formol-ether concentration technique (microscopic examination) was used to detect eggs of *Taenia* species as well as other parasites in stool. Confirmation of *T. solium* taeniosis was done by detection of adult *T. solium* specific antibodies in sera of participants tested positive by copro-Ag-ELISA using enzyme-linked immunoelectrotransfer blot (EITB) assay (rES38) as described by [24].

### CT scan

All participants with a history of epileptic seizures and seropositive by Ag-ELISA (n = 28) and one-fourth of Ag-ELISA seropositive participants without a history of epileptic seizures (n = 27), selected at random, were referred to Vwawa District hospital in Mbozi district, Mbeya and then transported to Muhimbili National Hospital in Dar es Salaam to be examined for evidence of NCC using computed tomography (CT) scan conducted with intravenous contrast. An image series of slides was made for each patient both before and after the contrast injection which were all examined by the same radiologist.

### Data management and analysis

All data were initially entered into EPI info software version 6. For analysis, the data were converted to Statistical Package for Social Scientist (SPSS) version 13. Bivariate analysis was first performed by Chi-Square test to assess the marginal association between human cysticercosis seropositivity and factors such as age group, gender, religion, presence of latrine, source of drinking water, hand washing practice, pork consumption, boiling of drinking water, history of passing ploglottides, and being positive by copro-Ag-ELISA at a 95% confidence level. To assess potential associations, a multivariate logistic regression analysis was performed by calculating OR and 95% confidence intervals for human cysticercosis seropositivity (Ag-ELISA/Ab-ELISA) and identified risk factors. The Ag-ELISA or Ab-ELISA was entered as an outcome and the covariates that were included in the model were age group, gender, presence of latrine, source of water, hand washing practice and being positive by copro-Ag-ELISA.

## Results

By the end of the study 70 households dropped out such that 830 in total completed the survey (92.2% compliance). Out of 830 enrolled participants, 476 (57.4%) were males and 354 (42.6%) were females. The age range of study participants was 15–60 years with mean age of 37.9±11.3 (SD) years. A large proportion of participants had finished primary education (77.1%) and the least had college or university education (0.5%). Regarding participants' religions, Christians were the majority (82.4%), followed by no religion/pagans (15.9%), and Muslims (1.7%). Furthermore, inhabitants enrolled in the study consisted mostly of farmers (85.4%).

Of the 830 human sera examined, Ag-ELISA found 16.7% with active cysticercosis while Ab-ELISA detected an overall seroprevalence of 45.3%. Furthermore, among the 427 participants in total who tested positive serologically, 20.6% tested positive with both tests, while 11.9% tested positive with Ag-ELISA only and 67.5% tested positive with Ab-ELISA only (see Table 1).

**Table 1:** Prevalence of *Taenia solium* and other helminth infections in Mbozi district, Mbeya Region, Tanzania.

Disease and test applied	Sample size	Number pos	Percentage (95%CI)
**Cysticercosis**			
Ag-ELISA (B158/B60)	830	139	16.7 (14.2–19.2)
Ab-ELISA (rT24h)	830	376	45.3 (41.9–48.7)
*Results on parallel test with Ag-ELISA and Ab-ELISA*			
Pos Ag-ELISA/Pos Ab-ELISA	830	88	10.6 (8.5–12.7)
Pos Ag-ELISA/Neg Ab-ELISA	830	51	6.1 (4.5–7.8)
Neg Ag-ELISA/Pos Ab-ELISA	830	288	34.7 (31.5–37.9)
**Neurocysticercosis (CT-scan of selected Ag-ELISA pos)**			
Contrast CT scan	55	30	54.6 (51.2–58.0)
With history of seizures	28	28	100 (100–100)
Without history of seizures	27	2	7.4 (−3.54–18.34)
**Taeniosis**			
Copro-Ag-ELISA	820	43	5.2 (3.7–6.8)
EITB (rES38)	820	34	4.1 (2.8–5.6)
Microscopic examination	820	9	1.1 (0.4–1.8)
*Results on parallel test with Copro-Ag-ELISA and EITB (rES38)*			
Pos Copro-Ag-ELISA/Pos EITB	43	34	79.1 (76.3–81.9)
Pos Copro-Ag-ELISA/Neg EITB	43	9	20.9 (18.1–23.7)
*Other intestinal parasites detected on microscopic examination*			
Hookworm eggs	820	116	14.1 (11.7–16.5)
*Schistosoma mansoni* eggs	820	13	1.6 (0.8–2.5)
*Ascaris lumbricoides* eggs	820	8	1.0 (0.3–1.7)

Pos = Positive; Neg = Negative; 95%CI = 95% Confidence Interval.

Of the 820 study participants who returned stool samples, 5.2% tested positive for taeniosis by copro-Ag-ELISA, and 79.1% of copro-Ag-ELISA positive participants had *T. solium* adult worm specific antibodies (taeniosis) using EITB indicating 4.1% of total participants had *T. solium* specific taeniosis. In addition, *Taenia* spp. eggs were detected using formol-ether concentration technique (microscopic examination) in 1.1% stool samples. Other parasite eggs detected during coprological examination included those of hookworms (14.1%), *Schistosoma mansoni* (1.6%) and *Ascaris lumbricoides* (1.0%) (see Table 1).

Generally most households surveyed had pit latrines (98.4%), however, 66% of pit latrines in those households had partial walls making them easily accessible to free roaming pigs. In addition, 54% reported using unsafe sources of drinking water such as stagnant, surface water from streams, ponds, or uncovered shallow wells. Information on personal hygiene indicated that multiple people using the same water in which to wash their hands by dipping was the most common method (54.3%), while no one reported washing their hands with soap.

With regard to information on symptoms associated with human cysticercosis 14.8% acknowledged a history of epileptic seizures, 52.0% severe chronic headaches, 17.1% loss of consciousness, and 50.8% partial seizures. Only history of epileptic seizures and chronic severe headaches were determined to be statistically important symptoms associated with human cysticercosis (p≤0.05) (Table 2).

**Table 2:** Associations between reported neurological symptoms and *T. solium* positivity (Ag- and Ab-ELISA), Mbozi district, Tanzania.

	Total		Ag-ELISA pos			Ab-ELISA pos		
Symptoms	Number	%	Number	%	*P-value*	Number	%	*P-value*
*Epileptic seizures*								
Yes	123	14.8	28	22.8	0.050	66	53.7	0.044
No	707	85.2	111	15.7		310	43.8	
*Chronic severe headache*								
Yes	431	52.0	83	19.3	0.050	202	46.9	0.306
No	397	48.0	56	14.1		174	43.1	
*Loss of consciousness*								
Yes	142	17.1	23	17.1	0.847	65	45.8	0.901
No	688	82.9	116	16.9		311	45.2	
*Partial seizures*								
Yes	422	50.8	67	15.9	0.495	193	45.7	0.799
No	408	49.2	72	17.6		183	44.9	

Pos = positive.

Neurocysticercosis was detected by cranial CT scan in 30 (54.6%) of the 55 Ag-ELISA seropositives who accepted to undergo examination. All of the 28 seropositive people with a history of epileptic seizures were CT-scan positive for NCC. Twenty-eight of the thirty NCC cases detected by CT scan (93.3%) had more than 5 cysts in the brain.

### Multiple regression analysis

The risk factors that were significant in the analysis as risks associated with human cysticercosis are presented in Table 3. Being older age was found to be a risk for seropositivity with human cysticercosis in the regression model, as the Ag-ELISA was higher in the age groups 36–45 years (OR = 2.5) and 46–60 years (OR = 2.6) as compared to young people (<25 years). In addition, those who reported washing their hands by the dipping method using the same water as others were more likely to be seropositive by Ag-ELISA (OR = 3.8).

**Table 3:** Multivariate analysis on risk factors for human cysticercosis considered in the logistic regression analysis.

Risk factor	OR (95%CI)	*P-value*
*Risk factors associated with active human cysticercosis (Ag-ELISA pos)*		
**Age**		
15–25 years	Ref	0.02
26–35 years	1.8 (0.9–3.8)	
36–45 years	2.5 (1.2–5.0)*	
46–60 years	2.6 (1.3.5.3)*	
**Hand washing method**		
Running water	Ref	0.0001
Dipping	3.8 (2.5–5.9)*	
**Confirmed tapeworm carrier by copro-Ag-ELISA**		
Neg copro-Ag-ELISA	Ref	0.03
Pos copro-Ag-ELISA	2.6 (1.3–5.2)*	
*Risk factors associated with exposure to larval T. solium (Ab-ELISA pos)*		
**Sex**		
Male	Ref	0.005
Female	1.6 (1.1–2.1)*	
**Hand washing method**		
Running water	Ref	0.001
Dipping	0.2 (0.1–0.3)*	
**Confirmed tapeworm carrier by copro-Ag-ELISA**		
Neg copro-Ag-ELISA	Ref	0.05
Pos copro-Ag-ELISA	2.6 (1.3–5.2)*	
Non response	0.9 (0.2–3.7)	
**Source of water**		
Safe	Ref	0.0001
Unsafe	1.9 (1.4–2.5)*	

OR = odds ratio; 95%CI = 95% confidence interval; Ref = reference; pos = positive; neg = negative.

* Significant factor at 95%CI.

On the other hand, when fitting the risk factors for seropositivity with human cysticercosis by Ab-ELISA in the regression model, results showed the risk for seropositivity to be significantly higher for males as compared to females (OR = 1.6), and for people using an unsafe source of water (OR = 1.9). Furthermore, being confirmed a *T. solium* tapeworm carrier by copro-Ag-ELISA substantially increased the risk for being seropositive for cysticercosis by both tests.

## Discussion

This is the first community-based study in Tanzania to determine the magnitude of human *T. solium* infection in an area previously reported to have a high prevalence of porcine cysticercosis. Findings indicate detection of active cysticercosis in 16.7% of the human population while 45.3% tested positive for exposure to larval *T. solium* through detection of antibodies. These findings are higher in comparison to that reported elsewhere in the region such as 4.6% (Ag-ELISA), 6.9% (EITB) and 40.8% (Ab-ELISA) in Burundi [25], 12.1% (Ab-ELISA) in Mozambique [26], 0.4%–3% (Ag-ELISA) in Cameroon [27], [28], and 7.7% (Ag-ELISA and EITB) in Senegal [29], but lower than the 21.6% (Ag-ELISA) recently reported from the Democratic Republic of Congo [30].

Of the study participants who reported a history of epileptic seizures 22.8% were seropositive by Ag-ELISA (all these were also CT-scan positive for NCC) while 53.7% were positive by Ab-ELISA. These findings are higher in contrast to another study in Africa which used both tests whereby 1.2% and 44.6% of people having epileptic seizures were found positive by Ag-ELISA and Ab-ELISA, respectively, in Cameroon [31]. The findings on antibody detection in people having epileptic seizures were higher in this study compared to other reports elsewhere: 14% (EITB) in northern Tanzania [14], 35.7% (Ab-ELISA) in western India [32], 22.5% (EITB) in Peru [33] and 31.3% (EITB) in Mexico [34]. These results are of course influenced by the method or definition applied for epileptic seizures. Furthermore, the results are in agreement with findings in other studies indicating a strong association between epileptic seizures and cysticercosis [5], [35], [36], [37]. Indeed a recent community-based survey of adult active epilepsy in northern Tanzania [38] identified a high rate of focal onset seizures suggesting that a high proportion of epilepsy in the studied population may be due to identifiable, and possibly preventable, causes specifically referring to NCC and recommended further investigation of risk factors.

Three tests were used to diagnose taeniosis due to *T. solium* for prevalence estimation: the formol-ether concentration technique for detecting *Taenia* spp. eggs, the copro-Ag-ELISA for detecting antigens of *Taenia* species tapeworms [39] and the EITB-(eRS38) for detecting antibodies to adult *T. solium* tapeworms. The overall prevalence of taeniosis of 1.1% by formol-ether concentration technique, and 5.2% by copro-Ag-ELISA, are within the previous reported range of 0.6%–1.9% (microscopy) and 2.8%–14% (copro-Ag-ELISA) from comparable studies in Mexico and Peru [33], [40], [34]. These results also support previous findings that the prevalence of intestinal taeniosis is low in most endemic communities and microscopy-based figures underestimate the true prevalence of *T. solium* and would be a poor monitoring tool for control purposes. The detection of 4.1% *T. solium*-specific taeniosis cases in this study using EITB (eRS38) is substantial though we are unable to compare these findings to other surveys in Africa since, to the authors' knowledge, no other studies reporting the use of EITB for detection of *T. solium* tapeworm antibodies have been reported from the continent. However, further development of a simple and highly specific test for detecting *T. solium* taeniosis is warranted as the sensitivity of the EITB for taeniosis is reported to be 94.5–97% and there are reports of cross reaction of eRS38 to *T. saginata* and *Schistosoma* species [24], [41].

Consumption of *T. solium* cysticerci in pork is crucial for transmission of human *T. solium* infections (taeniosis/cysticercosis). Pork consumption was the only significant risk factor associated with NCC found among epileptic patients studied in northern Tanzania [42]. Home slaughtering of pigs with only limited or no inspection of the carcass/meat as well as frequent consumption of undercooked pork at local brew bars have been noted to be common practices in the southern highlands [43]. Community-based studies on porcine cysticercosis conducted in Tanzania have reported high prevalences of porcine cysticercosis in different parts of the country where pig keeping is popular: 17.4% (lingual exam) in the northern highlands district of Mbulu [11], 5.1%–16.9% (lingual exam) and 30.4%–32.0% (Ag-ELISA) in districts of Iringa, Mbeya and Ruvuma regions in the southern highlands [12]. As shown in Mbozi district infected pigs can be a sentinel of human infections with *T. solium* thus these other areas are likely to be endemic for human cysticercosis and taeniosis as well.

From Tanzania [12] reported increasing production and transportation of pigs from endemic rural communities where cysticercosis control regimens are limited or non-existent to large urban areas where pork demand is high and growing. Transportation of pigs and pork from rural cysticercosis endemic areas increases the risk of transporting infected and diseased pigs/pork into non-endemic urban areas for slaughter/sell as evidenced by [13] who found 5.9% of pigs brought into Dar es Salaam for slaughter were infected with *T. solium* by routine meat inspection. This has important epidemiologic implications for the country regarding transmission of cysticercosis/taeniosis especially in urban areas where pig keeping is not practiced but pork consumed.

Porcine cysticercosis has also been reported in many of the countries bordering Tanzania including Kenya, Uganda, Zambia, Democratic Republic of Congo and Mozambique [44], [45], [46], [47], [48]. These results indicate widespread presence of *T. solium* infections in the region such that cysticercosis should be viewed as an eastern and southern Africa regional issue rather than as a Tanzania specific problem.

It has been found that sero-prevalence of active human cysticercosis in the studied communities showed an increase with age and reached a maximum in participants aged 36–60 years. This supports the finding among epilepsy patients in northern Tanzania with NCC who tended to be older with a later onset of seizures compared to those without NCC [42]. Similar observations were reported by [32] in western India and [49] in Chaco region, Bolivia. However, the age dependency was not observed with regard to detection of antibodies against cysticerci which has been shown by others [50] indicating the need for further studies on *T. solium* transmission dynamics in the area.

The prevalence of antibodies to cysticercosis in females was significantly higher than in males in this study which agrees with the findings reported by [40] in Guatemala showing higher infection proportions in females but contrasts with the findings reported by [51] in Vietnam which reported higher prevalence in males as well as the findings reported by [49] in Chaco region, Bolivia, which showed no significant difference between genders.

Strong association between taeniosis and human cysticercosis (both active and antibodies) was found in the present study similar to other studies conducted in different areas of the world [32], [33]. The significant numbers (34) of sera of the subjects diagnosed with active taeniosis by copro-Ag-ELISA were positive with both Ag-ELISA and Ab-ELISA. Similarly [33], [34], [51] found similar results that many study subjects in Peru, Mexico and Vietnam, respectively, were infected with both taeniosis and cysticercosis.

In [52] it was reported that cysticercosis may also occur in humans if eggs are conveyed to the mouth by unclean fingers after defecation or eating without washing hands. This study found significant association between the type of hand washing and human cysticercosis prevalence in contrast to the study by [53]. Washing with running water in contrast to dipping, may be due to a bias in the sense that availability of running water in the household reflects a different sanitary level in general.

Although the symptoms and signs of NCC are highly protean, it is commonly associated with late onset of epileptic seizures, focal neurological signs (partial seizures), severe chronic headache, intra-cerebral hypertension, and cognitive behavioral dysfunctions [33], [54], [55]. The frequencies of different manifestations, complications and disabilities associated with NCC estimated through a WHO-commissioned review found that among patients seen in neurology clinics, epileptic seizures were the most common manifestation (78.8%) followed by headaches (37.9%), focal deficits (16.0%) and signs of increased intracranial pressure (11.7%) [56]. High correlation was found in this study between history of epileptic seizures and seropositivity with Ag-ELISA and Ab-ELISA as well as CT scan results. All 28 participants with a history of epileptic seizures and seropositive by Ag-ELISA (as inclusion criteria for CT scan in this study) had NCC features on CT scan, and consequently at least 22.8% of participants reporting history of seizures had NCC, as mentioned earlier. This indicates a significant contribution of NCC to active epilepsy cases diagnosed in the current study. Nevertheless, this figure is higher than expected for developing countries. Using a questionnaire approach we cannot rule out false-positive answers, however if epileptic seizures were over-reported the proportion with confirmed NCC would actually go down. Similarly, severe chronic headache was an important factor associated with Ag-ELISA seropositivity, indicating increased intracranial pressure. Similar findings have been reported by [27], [32], [34].

Despite the fact that *T. solium* infections have been suggested to be successfully controlled in an endemic country like Mexico [57], the lack of diagnostic tools for detection of human cysticercosis/neurocysticercosis (e.g. serological tests, neuroimaging) and taeniosis (e.g. copro-Ag-ELISA, EITB) in Tanzania is a constraining factor for effective management and control of the infections at the community level. Moreover, the cost for the neuroimaging tests (CT scan or MRI) is too expensive for ordinary people and their availability is very limited in resource poor endemic areas. Detection of circulating *T. solium* cysticercosis antigen using Ag-ELISA has been shown to improve the diagnostic potential for neurocysticercosis, especially in areas where neuroimaging techniques are not accessible [58]. The establishment of affordable, reliable diagnostic tools as well as sustainable and appropriate preventive and control measures is warranted as the reduction of neurocysticercosis would be expected to significantly reduce the burden of epilepsy [59].

### Conclusion

Findings of the present study indicate that human *T. solium* infection is hyper-endemic in Mbozi district, Mbeya Region, Tanzania. Further understanding of the disease distribution and transmission burden in Tanzania is needed as well as strengthening capacity to address it. Correspondingly, cross-sectoral (stakeholders) control efforts at local, regional, national level as well as across endemic neighboring countries of the eastern and southern Africa region should be initiated using appropriate, sustainable approaches to decrease or eliminate the burden of the disease.

## Supporting Information

Text S1 **STROBE checklist.**
(DOC)
